# Obesity masks the relationship between dietary salt intake and blood pressure in people of African ancestry: the impact of obesity on the relationship between sodium and blood pressure

**DOI:** 10.5830/CVJA-2018-011

**Published:** 2018

**Authors:** Maseko Muzi, Mashao Mercy, Bawa-Allah Abdulraheem, Phukubje Edgar, Mlambo Bongubuhle, Nyundu Thamsanqa

**Affiliations:** School of Physiology, Faculty of Health Sciences, University of the Witwatersrand, Johannesburg, South Africa; School of Physiology, Faculty of Health Sciences, University of the Witwatersrand, Johannesburg, South Africa; School of Physiology, Faculty of Health Sciences, University of the Witwatersrand, Johannesburg, South Africa; School of Physiology, Faculty of Health Sciences, University of the Witwatersrand, Johannesburg, South Africa; School of Physiology, Faculty of Health Sciences, University of the Witwatersrand, Johannesburg, South Africa; School of Physiology, Faculty of Health Sciences, University of the Witwatersrand, Johannesburg, South Africa

**Keywords:** dietary salt intake, obesity, hypertension, salt retention, body mass index

## Abstract

Previous studies conducted to investigate the relationship between sodium intake and blood pressure in our African population have yielded contradictory results. With the high prevalence of obesity in this population, it is possible that these contradictory findings are due to the masking effects of obesity on this relationship. We measured 24–hour ambulatory blood pressure and 24–hour urine excretion on 547 South Africans of African ancestry. A multivariate regression analysis revealed no independent relationship between 24–hour sodium excretion and blood pressure in the total population sample, but when participants were stratified according to body mass index (BMI) status, there was a significant association between 24–hour sodium excretion and blood pressure in the normal–BMI participants but not in the overweight/obese participants. We concluded that dietary salt intake, indexed by 24–hour urinary sodium excretion, was associated with increased ambulatory blood pressure but this relationship was masked because of a high proportion of overweight/obese individuals in this population.

Cardiovascular diseases (CVD) are presently a leading cause of death in South Africa and sub–Saharan Africa (SSA).^1^,^2^ Hypertension remains the commonest risk factor for CVD.^3^,^4^ A number of studies have associated an increased dietary sodium (Na+) intake and obesity with hypertension and target–organ damage.^5^

In an effort to lessen the worldwide prevalence of hypertension, global strategies and population–based intervention studies, including Dietary Approaches to Stop Hypertension (DASH), have focused particularly on reduction of Na+ intake as a means of lowering blood pressure (BP) in a population.^6^–^8^ This could be of benefit to people of African descent because a number of studies have shown that the incidence of hypertension is increasing in black communities.^9^–^11^ However there is a gap in our knowledge on the role of dietary sodium on blood pressure in this community.

Even though previous studies conducted in this population have revealed a high prevalence of hypertension,^12^,^13^ the relationship between dietary salt intake and BP is still not well understood because studies have revealed contradictory findings on this relationship.^14^,^15^ One study showed a modest association,^16^ while others showed no association.^17^,^8^ In one study, the investigators showed an association between BP and the sodium–to–potassium ratio, which is also an index of dietary sodium intake, but they could not show any direct relationship between BP and dietary sodium.^18^

The contradictory findings of these studies are indicative of the complex relationship between BP and dietary sodium. In this population, the complexity of this relationship could be compounded by the high prevalence of obesity,^19^ as body mass index (BMI) has been shown to have a direct association with BP.^20^–^23^ A possibility exists that obesity masks the relationship between BP and dietary sodium intake in this population. The biggest contributor to the masking effect could be the high proportion of overweight or obese individuals, especially women. Therefore, in this study, in order to investigate whether the relationship between dietary salt intake (indexed by 24–hour urinary sodium excretion) and blood pressure is masked by obesity, we stratified participants according to BMI status.

## Methods

Informed consent was obtained from the participants, and the principles of the Declaration of Helsinki were adhered to. The study was approved by the University of the Witwatersrand Committee for Research in Human Subjects (approval number: M15–06–44) and forms part of the South African Hypertension and Diet Study (SAHDS), which is part of the ongoing African Project on Genes in Hypertension. The study design has been briefly described in other publications.^24^,^25^

We randomly recruited 1 219 South Africans of African ancestry from a metropolitan area of Johannesburg (Soweto). Of these participants, 547 (346 women and 201 men) were selected because they had complete 24–hour ambulatory BP (ABP) and 24–hour urine samples. The minimum age for the study participants was 18 years, and there was no upper age limit.

A standardised questionnaire was administered to each participant to obtain demographic data, medical history such as the presence of hypertension, and use of medication. Height and weight measurements were recorded with the participants wearing indoor clothes with no shoes. BMI was calculated as weight in kilograms divided by the square of height in metres.

Twenty–four–hour ambulatory BP monitoring was performed using oscillometric monitors (Spacelabs, model 90207). Standard cuffs with an inflatable bladder suitable for the participant’s arm circumference were used. The monitors were programmed to measure BP at 15–minute intervals from 06:00 to 22:00 and then at 30–minute intervals from 22:00 to 06:00. Participants kept a diary card for the duration of the recordings to note the time of going to bed in the evening and time of waking up in the morning. These times were used to determine the wake and sleeping periods. Participants also recorded the time when they took medication, smoked, drank caffeine or alcohol, depending on which was applicable to each participant. On completion of the recordings, data were transferred to a computer for analysis. Ambulatory blood pressure data were expressed as 24–hour average systolic and diastolic BP.

Timed urine samples were collected over a 24–hour period. Each participant was issued with a urine collection bottle and the bottles were then collected from each participant after 24 hours. Twenty–four–hour urine Na+ excretion rate was calculated from the product of urine volume and urine electrolyte concentration. Creatinine clearance was determined from the product of urine volume and urine creatinine concentrations divided by plasma creatinine concentration.

The quality of urine samples was determined by constructing regression relations between 24–hour urine creatinine and body weight, and 24–hour urine volume and age in gender–specific groups. Based upon the 95% confidence intervals for each group, a 24–hour urine sample was considered acceptable if 24–hour urine creatinine (mmol) was > 3.5 and < 35 for males and > 3.5 and < 30 for females. Samples with urine volumes < 500 ml/day were also assumed to be incomplete urine collections. These are standard approaches and have been published on numerous occasions by other groups.

## Statistical analysis

For statistical analysis the SAS software, version 9.4 (SAS Institute Inc, Cary, NC), was used. Data are shown as mean ± SD and p < 0.05 was considered significant. To determine the independent relationship between 24–hour urinary excretion and 24–hour systolic and diastolic BP, a multivariate regression analysis was used. Confounders such as age, gender, BMI (as a continuous variable), alcohol consumption, smoking, the presence of diabetes and treatment of hypertension were included in the regression model.

## Results

Table 1 gives a description of the demographic, anthropometric, haemodynamic and general clinical characteristics of the participants in this study. They were divided into three groups: total sample, men and women. The characteristics include age, BMI, 24–hour ambulatory BP, 24–hour urinary Na+ excretion rates, alcohol consumption, smoking, diabetes status and hormone concentrations.

**Table 1:** General and clinical characteristics of the study population according to gender and BMI status

	*Total sample*			*Men*			*Women*		
*Parameters*	*All*	*Normala*	*Overweightb/Obesec*	*All*	*Normal*	*Overweight/Obese*	*All*	*Normal*	*Overweight/Obese*
Age (years)	45.3 ± 18.5	36.3 ± 18.3	50.4 ± 15.2	45.5 ± 19.9	38.2 ± 18.9	53.5 ± 18.0	45.1 ± 17.7	33.7 ± 17.4	48.9 ± 16.1
BMI (kg/m^2^)	29.1 ± 7.8	21.6 ± 2.1	34.4 ± 1.4	24.9 ± 5.0	21.2 ± 2.0	29.1 ± 4.1	31.5 ± 8.1	21.9 ± 2.2	34.6 ± 6.7
Hypertensive (%)	35.1	17.6	43.8	24.4	19.8	30.2	39.3	14.9	47.5
Diabetic (%)	14.3	7.2	16.9	12.9	9.4	16.8	13.9	4.6	16.9
Alcohol intake (%)	23.6	33.1	18.0	41.8	46.7	36.8	12.7	17.2	11.1
Smokers (%)	15.2	26.4	9.1	33.8	43.4	23.0	4.3	5.7	3.9
Na+ (mmol/day)	105.6 ± 78.4	108.9 ± 89.8		103.8 ± 72.1106.5 ± 74.7	115.7 ± 71.1	98.5 ± 77.9	105.0 ± 80.55	121.5 ± 99.4	99.5±68.6*
HbA1c (%)	6.2 ± 1.5	5.8 ± 1.0	6.3 ± 1.6	6.1 ± 1.7	5.8 ± 1.4	6.5 ± 1.9	6.2 ± 1.3	5.7 ± 0.3	6.3 ± 1.5
Renin (ng/dl)	36.4 ± 73.8	35.9 ± 75.8	34.8 ± 35.7	35.2 ± 52.2	33.1 ± 51.6	37.5 ± 53.0	37.0 ± 84.1	39.5 ± 98.4	31.0 ± 5.0
Insulin (mmol/l)	14.4 ± 16.6	10.8 ± 13.8	16.8 ± 18.9*	13.8 ± 19.3	9.8 ± 13.3	18.9 ± 23.1*	14.8 ± 14.8	13.1 ± 14.1	15.5 ± 15.0
Leptin (ng/ml)	24.2 ± 25.3	9.9 ± 12.4	43.0 ± 29.4*	8.5 ± 8.7	4.3 ± 6.9	12.2 ± 8.4*	35.0 ± 27.2	17.6 ± 13.9	40.5 ± 28.1*

HbA_1c_, glycated haemoglobin; BMI, body mass index; Na+, 24-hour urinary sodium excretion rate.

^a^Normal BMI is defined as < 25 kg/m^2^, boverweight is defined as BMI ≥ 25 < 30 kg/m^2^ and cobese is defined as BMI ≥ 30 kg/m^2^.

The mean age of the total population sample was 45.3 ± 18.5 years. There was no age difference between males and females (men 45.5 ± 19.9 and women 45.1 ± 17.7 years) with 63.2% of participants being women. The mean BMI of the group was 29.1 ± 7.8 and 71% of participants were either overweight or obese. When BMI was calculated according to gender, more women were in the overweight/obese category (75%) compared to men (47%). Thirty–five per cent (35%) of the total sample population was hypertensive, 23.0% consumed alcohol regularly, 15% smoked, and 14% had diabetes mellitus or an HbA1c > 6.1%.

There was no significant difference in urinary sodium excretion in the total sample and in men; however the overweight/obese women had significantly lower urinary sodium excretion rates. Both insulin and leptin levels were significantly higher in the overweight/obese individuals compared to the normalweight participants. Gender differences were observed in leptin concentrations. Compared to men, leptin concentrations were significantly higher in women. Table 2 gives the haemodynamic characteristics of the population. Both systolic and diastolic BP values were higher in the overweight/obese participants compared to the normal–weight participants.

**Table 2:** Haemodynamic characteristics of the study population according to BMI status

	*All participants*	*Normal BMIa*	*Overweightb/Obesec*
Total sample			
SBP24 (mmHg)	118.6 ± 14.9	114.9 ± 12.3	121.6 ± 16.1*
DBP24 (mmHg)	72.2 ± 8.5	71.3 ± 9.1	74.4 ± 10.6*
Women			
SBP24 (mmHg)	116.9 ± 98.1	109.6 ± 9.8	116.6 ± 15.1*
DBP24 (mmHg)	71.9 ± 9.9	68.4 ± 7.3	71.3 ± 9.9*
Men			
SBP24 (mmHg)	123.2 ± 18.1	119.8 ± 12.15	125.4 ± 15.7*
DBP24 (mmHg)	64.4 ± 11.6	63.2 ± 11.1	66.8 ± 12.6*

BMI, body mass index; SBP24, 24-hour ambulatory systolic blood pressure; DBP24, 24-hour ambulatory diastolic blood pressure.

^a^Normal weight is defined as < 25 kg/m^2^, boverweight is defined as BMI ≥ 25 < 30 kg/m^2^ and cobese is defined as BMI ≥ 30 kg/m^2^. *p < 0.05 vs normal BMI.

Table 3 shows the relationship between 24–hour urinary sodium excretion and 24–hour systolic and diastolic BP. There was no significant relationship between 24–hour sodium excretion rate and BP in the total sample but when participants were stratified according to BMI status, the relationship reached statistical significance in the normal–weight individuals, even though it remained insignificant in the overweight/obese participants.

**Table 3:** Relationship between dietary sodium intake and 24-hour ambulatory BP according to gender and BMI status

	*Total sample*			*Men*			*Women*		
All participants	*Partial r2*	*95% CI*	*p-value*	*Partial r2*	*95% CI*	*p-value*	*Partial r2*	*95% CI*	*p-value*
SBP24 (mmHg)	0.08	–0.01–0.16	0.0872	0.23	0.02–0.40	0.0252*	0.05	–0.06–0.15	0.4095
DBP24 (mmHg)	0.06	0.02–0.14	0.1469	0.21	0.02–0.40	0.0299*	0.07	0.03–0.18	0.1747
Normal BMIa									
SBP24 (mmHg)	0.11	0.02–0.19	0.0146*	0.25	0.05–0.43	0.0122*	0.07	–0.14–0.29	0.5016
DBP24 (mmHg)	0.10	0.01–0.18	0.0193*	0.29	0.10–0.47	0.0030*	0.06	–0.14–0.29	0.5016
Overweightb/obesec									
SBP24 (mmHg)	0.06	–0.04–0.17	0.2448	0.01	–0.2–0.19	0.9091	0.08	–0.05–0.21	0.2058
DBP24 (mmHg)	0.07	–0.04–0.17	0.2551	0.01	–0.2–0.21	0.9751	0.09	–0.02–0.22	0.1281

CI, confidence intervals; SBP24, 24-hour systolic blood pressure; DBP24, 24-hour diastolic blood pressure; BMI, body mass index.

^a^Normal BMI is defined as < 25 kg/m^2^, boverweight is defined as BMI ≥ 25 <30 kg/m^2^ and cobese is defined as BMI ≥ 30 kg/m^2^.

Adjustments were made for age, gender (in the total population), BMI (as a continuous variable), hypertension, diabetes, smoking and alcohol intake.

**Fig. 1:**
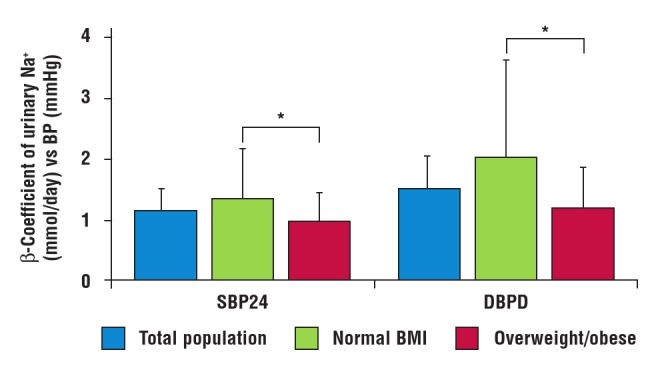
Multi–adjusted slopes (β–coefficients) of 24–hour urinary sodium excretion versus 24–hour systolic and diastolic blood pressure in the total sample, normal–weight and overweight/obese participants. Adjustments were made for age, gender, smoking, alcohol intake, diabetes and hypertension. BMI, body mass index; BP, blood pressure; SBP24, 24–hour systolic blood pressure; DBP24, 24–hour systolic blood pressure.

When participants were divided according to gender, there was a significant association between urinary sodium excretion rate and BP in the total sample of men and in the normal–weight men but not in overweight men. In women there was no relationship between 24–hour urine excretion and BP, irrespective of BMI status. Fig. 1 compares the slopes of the relationship between 24–hour urinary sodium excretion and BP in the total population. The slope (β–coefficient) of the normal–BMI participants was significantly higher than that of the overweight/obese group.

## Discussion

To determine the masking effects of an increased BMI, we first investigated the relationship between 24–hour urinary sodium excretion and BP in the total population sample. Consistent with previous studies in this population^4^–^6^ and in other population groups,^29^,^30^ we found no relationship. In spite of the inconclusive results in this population, the relationship between sodium level and BP has been well established in a number of studies,^31^–^34^ therefore our current findings require thorough scrutiny.

The limitations of previous studies conducted in this population could be that they did not take into consideration the high prevalence of obesity. So in order to account for the high incidence of obesity, in this study we stratified participants according to BMI status.

In a multivariate regression analysis, there was a statistically significant relationship between 24–hour urinary sodium excretion and both systolic and diastolic BP. However sodium level was not related to BP in the overweight/obese group. This difference was further demonstrated when the slopes of this relationship were compared between the two groups. The β–coefficients of the normal–BMI participants were significantly higher than those of the overweight/obese groups. This is indicative of a stronger relationship in the normal–weight group compared to the overweight/obese individuals.

The differences in the relationship between the two groups are indicative of the masking effects of obesity. These masking effects were further confirmed when the participants were divided according to gender. In men, the relationship was present in the total sample of men and in the lean group but not in the overweight/obese group. In the women, no relationship was observed irrespective of BMI status.

The gender differences are due to dissimilarities in the degree of obesity in the two groups. In men the average BMI was 24.9 ± 5.0 kg/m^2^ and only 47% were overweight or obese. On the other hand the average BMI in women was 31.5 ± 8.1 kg/m^2^ with 75% of the women in the overweight/obese category. Due to the lower BMI and lower proportion of overweight/obese men, the relationship between sodium and BP was not masked. On the other hand, the significantly higher BMI and higher proportion of overweight/obese women resulted in the masking of the relationship in the female group. Since women made up 63% of the total population sample, the masking effects of this female group are imputed to the whole population sample.

Mechanisms responsible for the masking effects seem to be mediated by leptin. Plasma renin and insulin levels were similar between the different groups but leptin was significantly higher in the overweight/obese group, especially in women. This could account for the significantly lower urinary sodium excretion rate in the overweight/obese women compared to normal–weight women. As explained earlier, women made up 63% of this population and 75% of them were overweight or obese, therefore they were responsible for the significantly lower urinary sodium excretion rate (106 mmol/day) in this population compared to other population groups.

In a study conducted in 45 countries with a total of 69 011 participants, the average 24–hour urinary sodium excretion rate was 159.4 mmol/day.^35^ In a Chinese study, the average 24–hour urinary sodium excretion was 235 mmol/day,^3^ which is more than double that of our population. A low sodium excretion rate of 104 mmol/day was observed in the trials of the Hypertension Prevention Collaborative Research Group.^36^ However in this study, the participants were on a low–sodium diet. The relatively low urinary sodium excretion rate is unique to our population sample and it may account for the leptin–mediated masking effects.

The significantly higher leptin concentrations in the overweight/obese groups, especially in women where it was more than three times higher than that of the overweight/obese males, mediated the masking effects. Normally sodium intake results in a slight increase in BP due to volume expansion. This causes an increased sodium excretion rate through pressure natriuresis. A steady state is reached where sodium intake is equal to sodium excretion. However in the presence of high leptin concentrations the steady state is not achieved.

Although leptin stimulates the sympathetic nervous system, it also increases nitric oxide concentration.^37^ Therefore, the pressor effects of the sympathetic nervous system are counteracted by the depressor effects of nitric oxide. Consequently there is no net change in BP. Without an increase in BP, pressure natriuresis is suppressed, resulting in a reduced urinary sodium excretion rate. This explains the significantly lower urinary sodium excretion rates in overweight/obese women and in the total population sample since women constituted 63% of this sample.

The low urinary sodium concentration leads to a narrow range of urinary sodium concentration. As a consequence, the relationship between 24–hour urinary sodium excretion and BP is blunted. The masking of a relationship due to low concentrations is called a regression dilution.^3^ To the best of our knowledge, this is the first study to show that increased BMI masks the relationship between 24–hour urinary sodium excretion and BP, and that leptin mediates the mechanisms responsible for the masking effect.

Potential limitations of our study are as follows; firstly, we collected once–off 24–hour urine samples and this does not account for the daily variation in dietary sodium intake. Although 24–hour urinary sodium excretion is an acceptable index of dietary sodium intake, the once–off 24–hour urine sample does not take into account the within– and betweenperson sodium intake variations. Secondly, the covariates such as alcohol intake and smoking were obtained through a self–reported questionnaire and therefore may not have been accurate. Thirdly, we did not use a dietary recall questionnaire to assess sodium intake. This could have been a useful tool for assessing the accuracy of 24–hour urinary sodium excretion as an index of dietary sodium intake. Finally, this was a cross–sectional study, therefore conclusions regarding cause and effect must be drawn with caution.

## Conclusion

This study indicates that dietary sodium intake influenced BP in this population, however this relationship could only be observed in lean participants, since increased BMI masked this relationship. These data suggest that future studies must take into account the high prevalence of obesity in this population before any conclusions are drawn on the relationship between sodium intake and BP.
